# Correlation between the Friedman Classification and the Apnea-Hypopnea Index in a population with OSAHS

**DOI:** 10.1590/S1808-86942010000500004

**Published:** 2015-10-22

**Authors:** Marcos Marques Rodrigues, Ralph Silveira Dibbern, Carla W. Kruel Goulart, Robson Antonio Palma

**Affiliations:** 1 Resident Physician - Department of Otorhinolaryngology - Santa Casa de Limeira; 2 MSc in Otorhinolaryngology - USP-Ribeirão Preto. Otorhinolaryngologist - Department of Otorhinolaryngology - Santa Casa de Limeira; 3 Resident Physician - Department of Otorhinolaryngology - Santa Casa de Limeira; 4 Resident Physician - Department of Otorhinolaryngology - Santa Casa de Limeira. Irmandade da Santa Casa de Misericórdia de Limeira

**Keywords:** sleep apnea, snoring, polysomnography, obstructive.

## Abstract

**Abstract:**

OSAHS is an important disease in current medical settings because of its association with chronic cardiovascular diseases and socioeconomic impacts.

**Aims:**

to establish the correlation between the Friedman Classification and the OSAHS severity through the Apnea-Hypopnea Index) in patients with OSAHS for assessment and surgery purposes.

**Materials and Methods:**

Cross-sectional study. We evaluated and classified 143 patients, using the Epworth's and Friedman's scale and OSAHS severity according to the AHI.

**Results:**

112 patients were submitted to polysomnography. We compared the Friedman Score and the IAH. The variables were associated with p<0.05. We found that patients with light OSAHS, tended to have a lower Friedman classification (better surgical result). By the same token, patients with moderate to severe OSAHS had a greater prevalence of Friedman's score of III and IV; p< 0.05 (lower success rates from uvulopalatopharyngoplasty).

**Conclusion:**

We conclude that the Friedman's classification correlates with OSAHS severity - the higher the Friedman's score, the more severe is the OSAHS.

## INTRODUCTION

SOAS (Sleep Obstructive Apnea Syndrome), described in 1973 by Cristian Guilleminault^1^, is an important disease in current medical settings. It affects approximately 5% of the population^2^; however, it is estimated that it is a highly underdiagnosed disease^3^. It is relevant because of its correlation with chronic cardiovascular diseases which cause important morbid-mortality to those who have it, such as coronary disease^4^, stroke^5^, dyslipidemia and diabetes mellitus^5^.

The physiopathology of OSAS is still unclear. During apnea events there is a drop in oxygen saturation, causing the activation of pressure-reflexes, triggering a widespread activation of the sympathetic autonomous nervous system. There is an adrenergic flow causing peaks of tachycardia and hypertension. This process repeats many times during sleep in apneic patients and, in the long run, it causes high sensitivity of the peripheral chemoreflex, with an exaggerated response, even during normoxia, pressurereflex dysfunction, increase in adrenergic burden, long term cardiovascular dysfunction, systemic inflammation and metabolic deregulation with insulin resistance and type II diabetes mellitus^6^.

SOAS involve the obstruction of the upper airways (UAW) during sleep, causing apnea events with respiratory distress, being extremely important to establish the point of UAW obstruction in the assessment of patients with SOAS. In 1981, Fujita et al. created a classification for the UAW obstruction point^7^ during nasofibroscopy. We created a classification based on the Muller's maneuver in order to assess the airway obstruction in the oropharynx, tongue base and hypopharynx. Although the classification is old and highly criticized, it is still being used.

More recently, the Friedman classification has been used to assess oropharynx obstruction, assessing tonsil size, the Modified Mallampati Classification and the BMI varying between 1 and 4. This classification has been broadly used in most of the snoring and sleep apnea wards. It has a well-defined prognostic value in the literature: the higher the Friedman classification, the lower the success of uvulopalatopharyngoplasty^8^.

## OBJECTIVES

To establish the correlation between the Friedman Classification and SOAS severity by means of the HAI (hypopnea and apnea index) in order to assess and treat patients with sleep apnea.

## MATERIALS AND METHODS

This study was approved by the Ethics in Research Committee, under protocol # 113/08 and it was duly registered in the Clinical Trials Registry under ID NCT00883025. 143 patients with complaints of snoring and clinical history suggesting sleep apnea and symptoms of daily sleepiness, non-resting sleep and snoring were assessed in the SOAS Otorhinolaryngology Ward. All the patients were submitted to an assessment protocol, including interview, Epworth's scale, snoring subjective scale, Friedman's classification and complete otolaryngological exam (Table 1).

**Table 1:** Distribution of the Variables used in the SOAS protocol.

	STANFORD	BMIa	EPWORTH	AGE
Mean	8.16	29.42	11.25	47.95
Standard Deviation	2.10	5.55	5.14	11.25
Minimum	2	16.44	0	17
Maximum	10	51.02	24	77

^a^ Body Mass Index

The Friedman's classification is used to assess: palatine tonsils; modified Mallampati score and BMI (body mass index). This way, they are classified in four stages (I, II, III and IV). Patients with the lower Friedman's stage have a greater chance of success after uvulopalatopharyngoplasty in the treatment of SOAS^9^. Friedman's classification is shown on Table 2.

**Table 2:** Friedman's Classification

Stages	Modified Mallampati	Pharyngeal Tonsil	BMI
	1	3.4	<40
	2	3.4	<40
	1.2	1.2	<40
Stage II	13.4	3.4	<40
	3	0.1.2	<40
Stage III	4	0.1.2	<40
Stage IV	1.2.3.4	0.1.2.3.4	>40

The patients were submitted to nasofibroscopy with a 2.8mm Mashida scope. The test was done with the patient awake in the ward. We did not use vasoconstricting agents to assess the nasal cavity. After nasal cavity analysis, we did pharyngoscopy and a dynamic laryngoscopy.

All the patients were referred to polysomnography in the sleep lab. The participants were submitted to nocturnal polysomnography and full monitoring of the following channels: eye movements, leg movements, nasal airflow, chest movement, EEG, EKG, heart rate and oxygen saturation. The patients were broken down according to disease severity, following the criteria agreed upon by the American Sleep Association Task Force1. We took off the study those patients with craniofacial changes.

## RESULTS

**Graph 1:**
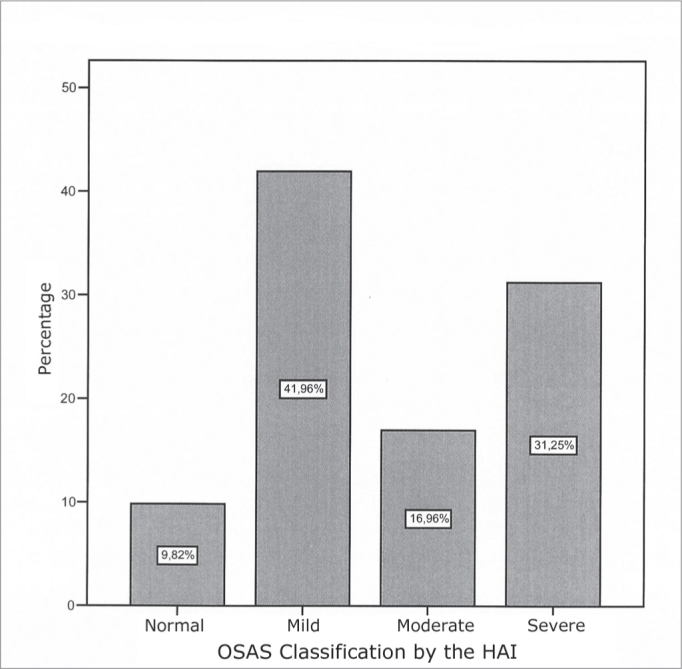
SOAS - NORMAL - HAI BELOW 5 ev/h MILD - HAI BETWEEN 5 AND 15 ev/h MODERATE - AIG BETWEEN 15 AND 30 ev/h SEVERE HAI GREATER THAN 30 ev/h

Of the 143 patients in the protocol, 112 were selected for the study and 31 were taken off the study for matching the exclusion criteria: not having a polysomnography study or for having an incomplete protocol. Distribution as to disease severity, in accordance to the criteria established by the American Sleep Academy Task Force1 is depicted on Graph 1.

**Graph 2:**
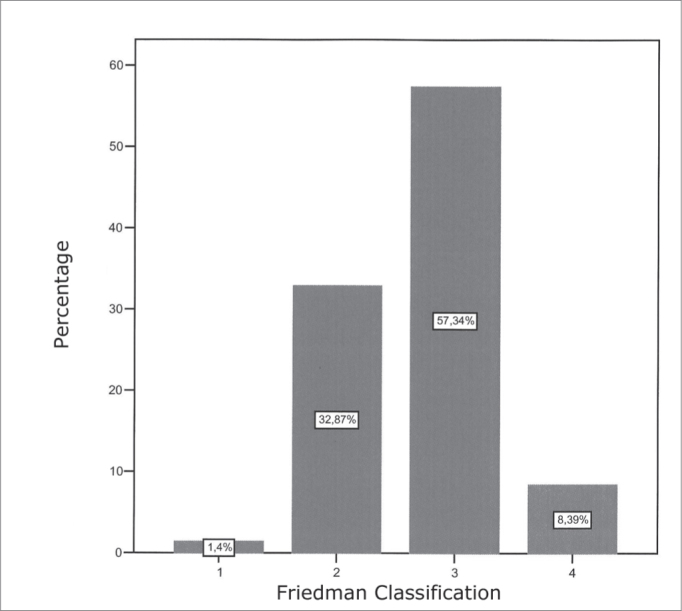
FRIEDMAN'S CLASSIFICATION 1 - FRIEDMAN I 2 - FRIEDMAN II 3 - FRIEDMAN III

All the patients in the initial evaluation were assessed and classified according to the Friedman classification, which results are shown on Graph 2.

SOAS classification and the Friedman classification were assessed in graphic cross-plotting and chi-squared statistical tests, with significant statistical correlation with the chi-square test by means of the linear association with p < 0.05. Result acquired by the analysis of the SSPS version 12.0 statistical software.

## DISCUSSION

The literature shows numerous surgical treatments for SOAS. Most of the literature studies show that the rate of improvements of HAI is between 45 and 90% with uvulopalatoplasty or pharyngoplasty as described by Hicklin et al.^10^. Jones et al., described an initial improvement rate around 34% and about 24% of immediate surgical failure^11^.

It is important to notice that there are only a few studies in the literature using only one type of surgery. In the study carried out by Jones et al. there are 151 cases reported, however with three types of surgical procedures, without mentioning a specific protocol to screen patients for surgery^12^.

**Graph 3:**
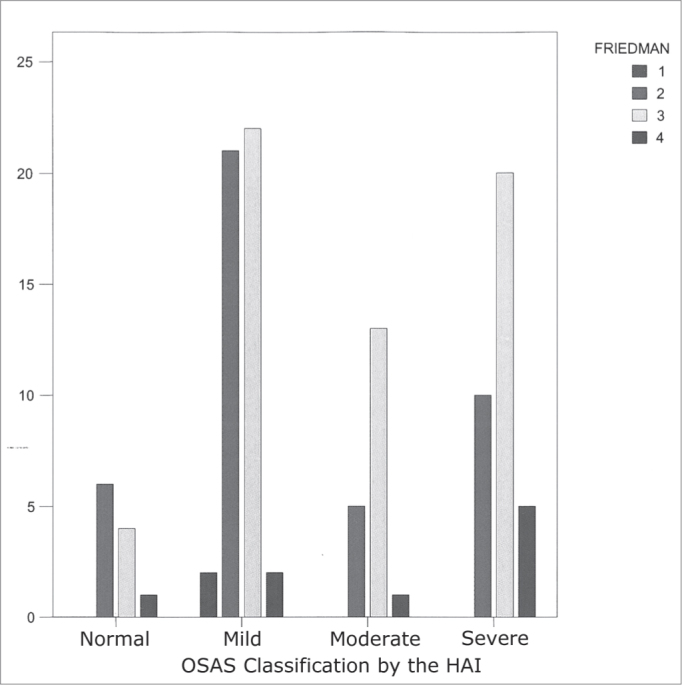
GRAPHIC CROSS-PLOTTING BETWEEN HAI AND FRIEDMAN

SOAS is a disease of airway obstruction with neuromuscular alterations, and a surgical approach must be indicated in order to correct the specific obstruction point and then obtain better results. For this reason, we carried out an association study between Friedman's classification and HAI. We considered important parameters in the evaluation of patients with SAOS. Video-nasofibroscopy is very important in the evaluation of the obstructive sites in the upper airways^7^. Friedman's classification was proven efficient in the prediction of the surgical success in the treatment of SOAS, as shown by Friedman et al.^12^.

We found a relationship in which patients with mild SOAS tend to have a lower Friedman score and, therefore, better surgical results^12^. By the same token, the patients classified with moderate to severe SOAS have a higher prevalence of the III and IV Friedman's classification, being associated with the lower success rates of uvulopalatopharyngoplasty (p<0.05).

The best results are obtained through a prior study, and patients must be always informed of their chances for improvement and the side-effects of uvulopalatopharyngoplasty - soft palate failure and dry mouth^13^.

Prior patient study is of easy clinical applicability. Friedman recently published a study showing that the Friedman classification has a high inter-examiner correlation, being useful to assess patients in research and teaching settings^14^.

These results point to the importance of a proper UAW investigation in the pre-op evaluation of all patients with SOAS. Patients must be assessed considering a series of variables. Friedman et al. showed that the Friedman classification is better than HAI in the prediction of uvulopalatopharyngoplasty success^11^. As seen in our sample, the Friedman classification is directly correlated with SOAS severity and must be assessed in the planning of surgical and/or clinical treatment.

## CONCLUSION

SOAS is a disease which mainly affects the upper airways with numerous intrinsic and extrinsic factors associated to its appearance. We then conclude that the Friedman's classification is correlated with HAI. The higher the Friedman score, the greater tends to be the apnea severity. This correlation is extremely useful in the preoperative evaluation of patients with SOAS concerning surgical indication criteria, and long term follow up is necessary.
